# Chromosome-level genome assembly of the large carpenter bee *Xylocopa dejeanii* Lepeletier, 1841 (Hymenoptera: Apidae)

**DOI:** 10.1038/s41597-025-05641-1

**Published:** 2025-07-23

**Authors:** Dan Zhang, Jianfeng Jin, Zeqing Niu, Michael C. Orr, Feng Zhang, Rafael R. Ferrari, Qingtao Wu, Qingsong Zhou, Wa Da, Chaodong Zhu, Arong Luo

**Affiliations:** 1 State Key Laboratory of Animal Biodiversity Conservation and Integrated Pest Management, Institute of Zoology, Chinese Academy of Sciences, Beijing, P.R. China; 2 Characteristic Laboratory of Forensic Science in Universities of Shandong Province, Shandong University of Political Science and Law, Jinan, P.R. China; 3 College of Life Sciences, Xinyang Normal University, Xinyang, P.R. China; 4 Department of Entomology, College of Plant Protection, Nanjing Agricultural University, Nanjing, P.R. China; 5 Environmental Science Training Center, Federal University of Southern Bahia, Porto Seguro, Brazil; 6 Tibet Plateau Institute of Biology, Xizang, P. R. China; 7 International College, University of Chinese Academy of Sciences, Beijing, P.R. China; 8 College of Biological Sciences, University of Chinese Academy of Sciences, Beijing, P.R. China

**Keywords:** Taxonomy, Genetics

## Abstract

Xylocopinae, a diverse bee subfamily comprising over 1,000 bee species, and also a major model system for studying the pollination and evolution of sociality. The lack of chromosome-level genome assembly resources for the Xylocopinae limits our research of their biology and evolution. Here, we provided the first pseudo-chromosomes genome assembly of the *Xylocopa dejeanii* combined PacBio CLR long reads, Illumina sequences, and Hi-C data. The final genome is 194.44 Mb located in 16 chromosomes. Our assembly includes 141 scaffolds, with a scaffold N50 length of 13.15 Mb. BUSCO analysis revealed 99.00% completeness. Genome annotation identified 28.27 Mb of repetitive elements, 10,970 protein-coding genes, and 432 ncRNAs. This high-quality *X. dejeanii* assembly advances our understanding of Xylocopinae genomics and provides new insights into bee evolution.

## Background & Summary

Bees are the most ecologically important group of pollinators, comprising over 20,000 described species worldwide^1^, but much work remains to be done regarding their genomics^2^. Among bees, the family Apidae, particularly the subfamily Xylocopinae, contains major pollinators of commercial crops, such as cardamom, passionfruit^3^, clover and more^4,5^. The carpenter bees of the genus *Xylocopa* Latreille, the largest genus within Xylocopinae, encompass nearly 400 recognized species found on all continents but Antarctica^4,6^.

Xylocopinae overall exhibits an exceptionally high variety of social behavior comparable to the diversity observed in Halictidae, making them an excellent model system for studying the evolution of sociality^7^. This subfamily spans the entire pectrum of social behaviors, from solitary bees such as those in the genus *Manuelia*^8^ to eusocial groups found in the tribe Allodapini^9,10^. Some xylocopine present cooperatively breeding, while others display a unique strategy characterized by prolonged maternal-offspring associations and communal overwintering of males and females within family units (namely in *Xylocopa*).

Despite their ecological and evolutionary significance, high-quality chromosome-level genomic resources are insufficient for Xylocopinae. To date, only eight assemblies exist for the >1,000 species of Xylocopinae, with only one for the genus *Xylocopa* (*Xylocopa violacea* [GCA_963969225.2]) (accessed on April 2025 from the NCBI database). Currently, the absence of high-quality chromosome-level genome resources significantly limits our understanding of the evolutionary history and social behavior of Xylocopinae, and also for bees in general.

In t Xylocopinae his study, we present the first high-quality pseudo-chromosomes genome of *Xylocopa dejeanii* Lepeletier, 1841, a species distributed across the southern Palaearctic and northern Indo-Malayan regions. Using Illumina sequencing, PacBio long reads, and Hi-C data, we assembled a genome of 194.44 Mb located across 16 chromosomes, comprising 141 scaffolds, with an N50 length of 13.15 Mb and, GC content of 43.50%. Our annotation predicted 10,970 protein-coding genes, 432 ncRNA genes, and 28.27 Mb repeat elements. As a high-quality pseudo-chromosomes assembly for the genus *Xylocopa*, this genome resource provides a critical foundation for comparative genomic studies on bee social behavior and pollination ecology within Xylocopinae.

## Methods

### Sampling and sequencing

All samples of *X. dejeanii* were collected in Motuo County, Linzhi, Xizang, China (E95.2889, N29.3238, 771 m) on July 20, 2020 by D.Z. and Q.W. All samples were immediately flash-frozen in liquid nitrogen, and then stored at −80 °C before subsequent DNA extraction in the lab. The bees were identified by Z.N., and all voucher specimens were deposited in the Institute of Zoology, Chinese Academy of Science. Prior to DNA extraction, we removed the metasomas of all individuals to minimize potential contamination from gut microbes. We used one male sample for PacBio, Illumina whole genome, Hi-C and Illumina transcriptome sequencing.

DNA extraction and sequencing were conducted by Berry Genomics (Beijing, China). PacBio, Illumina whole genome, Illumina transcriptome, and Hi-C sequencing were each performed using a single male individual. Genomic DNA for PacBio and Illumina whole-genome sequencing was done with a Qiagen Blood and Cell Culture DNA Mini Kit. The PacBio Sequel II platform was used for PacBio sequencing with 30 kb insert size sequencing libraries. For Illumina sequencing, a Truseq Nano DAN HT sample preparation Kit (Illumina USA) was used to generate sequencing libraries of 150 bp paired-end reads with an insert of 350 bp on the Novaseq6000 platform. Total RNA was extracted using TRIzol, and RNA libraries were prepared using a TruSeq RNA v2 kit. Hi-C sequencing was conducted on the BGI MGISEQ-2000 platform with 150 bp paired-end reads. In total, 88.98 Gb clean reads were generated, comprising 16.05 Gb (~82.56x) of Illumina whole-genome reads, 31.25 Gb (~160.70x) of PacBio reads, 34.37 Gb (~174.85x) of Hi-C reads, and 7.31 Gb RNA sequencing (Table 1).

**Table 1:** Statistics of the sequencing data used for genome assembly.

Libraries	Insert sizes (bp)	Clean data (Gb)	Sequencing coverage (x)
Illumina	350	16.05	82.56
PacBio	30 Kb	31.25	160.70
Hi-C	350	34.37	174.85
RNA	350	7.31	—

### Genome assembly

**Fig. 1:**
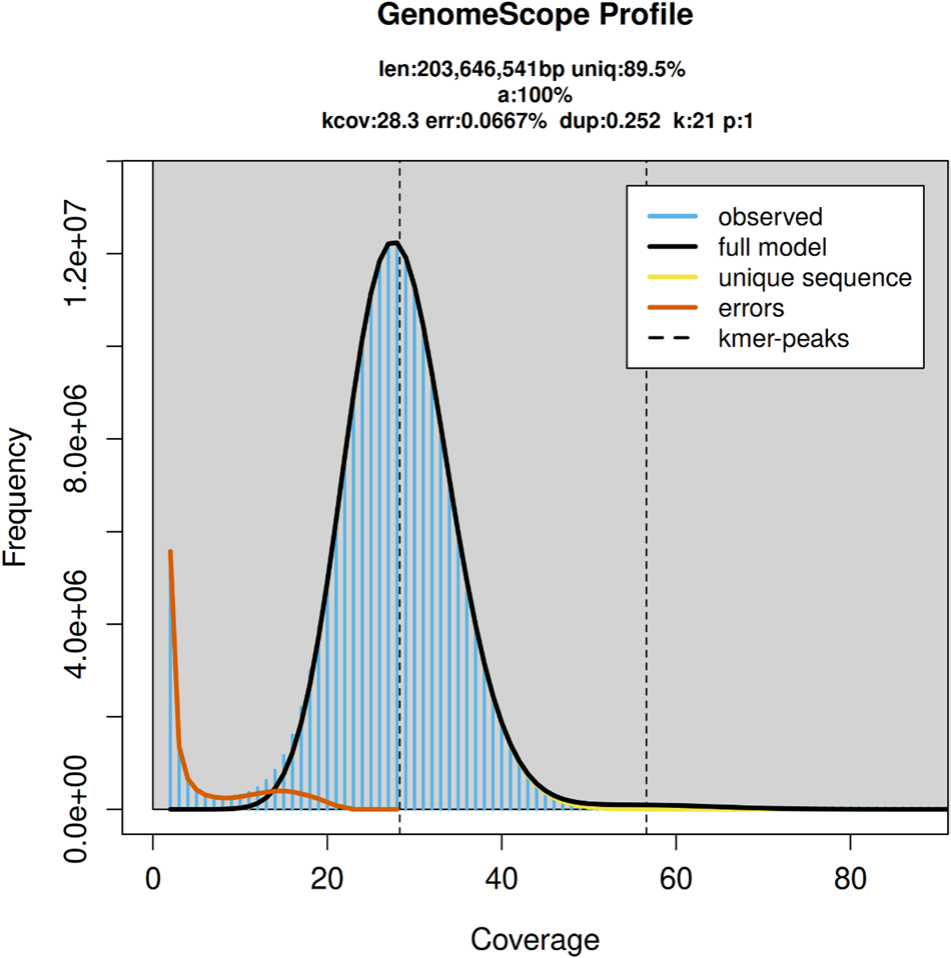
GenomeScope genome size estimates for *Xylocopa dejeanii*.

BBTools v38.29^11^ was performed for quality control: duplicate reads were removed by clumpify.sh, and low-quality reads were filtered with bbduk.sh. K-mer and k-distribution analyses were performed with khist.sh, and Genomescope v2.0^12^ was then used to estimate the size, repeat content, and heterozygosity of the *X. dejeanii* assembly, using a maximum k-mer coverage of 1,000. The genome survey estimated the genome size of *X. dejeanii* to be 203.65 Mb, based on the 21 k-mer frequency distribution of short reads (Fig. 1).

PacBio long reads were assembled via Flye v2.9^13^ using a minimum overlap between reads of 3000 bp and one round of self-polishing (“-m 3000 -i 1”). NextPolish v1.3.0^14^ was performed to polish the primary assembly with two rounds of short Illumina reads. Minimap2 v2.23^15^ was conducted to align the Illumina reads and PacBio assembly. Juicer v1.6.2^16^ was performed to Hi-C reads for alignment and quality control. Using 3D-DNA^17^ with default parameter, the primary contigs were anchored into chromosomal assembly. Juicebox v1.11.08^16^ was employed to correct potential errors. MMseqs2^18^ was used to identify potential contaminants by comparing sequences against the UniVec and BCBI nucleotide databases, with the “-min-seq-id” parameter, and sequences exhibiting more than 90% alignment were subsequently removed. BUSCO v5.4.4^19^ was run to assess genome completeness with the insecta_odb10 (n = 1,367) database as the reference.

**Fig. 2:**
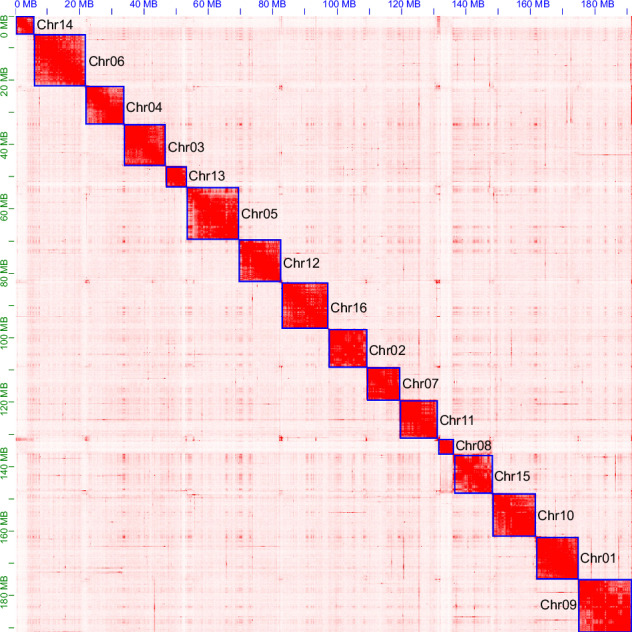
Genome-wide chromosomal heatmap of *Xylocopa dejeanii*.

Raw PacBio data were assembled with Flye, resulting in a 196.59 Mb assembly with a scaffold N50 length of 1.61 Mb in length (Table 2). After contaminant removal, polishing, and redundancy check, the final *X. dejeanii* assembly achieved a length of 194.44 Mb, including 141 scaffolds and 499 contigs, and the N50 length of 13.15 Mb, and GC content of 43.50% (Tables 2, 3), similar to others published bee genome sizes^20–22^. Among them, 191.64 Mb (98.54%) of the genome were anchored into 16 pseudo-chromosomes (Fig. 2). The BUSCO completeness of the final genome assembly is 99%, including 1,347 single-copy orthologs (98.50%), seven duplicate BUSCOs (0.5%), two fragmented BUSCOS (0.1%), and 11 missing BUSCOs (0.90%) (Table 2). The mapping rates of PacBio, Illumina, and RNA reads to the final assembly, and the mapping rates were 99.52%, 97.94%, and 91.80%, respectively. The low proportion of duplicated and missing BUSCOs, combined with high mapping rates of raw sequencing data and outstanding assembly metrics, underscores the remarkable contiguity and completeness of our genome assembly.

**Table 2:** Genome assembly statistics for *Xylocopa dejeanii*.

Assembly	Total length (Mb)	Number scaffolds/contigs (chromosomes)	Scaffold/contig N50 length (Mb)	GC (%)	BUSCO (n = 1,367) (%)			
					C	D	F	M
Flye	196.59	1,414/1,424	1.61/1.55	43.48	99	0.5	0.2	0.8
NextPolish	196.58	1,414/1,424	1.61/1.55	43.48	99	0.5	0.1	0.9
3D-DNA	195.36	303/661	13.15/1.43	43.50	99	0.5	0.1	0.9
Final	194.46	141/499	13.15/1.46	43.50	99	0.5	0.1	0.9

Complete BUSCOs (C); Complete and single-copy BUSCOs (S); Complete and duplicated BUSCOs (D); Fragmented BUSCOs (F); Missing BUSCOs (M).

**Table 3:** Genome assembly and annotation statistics for the chromosome-level assemblies of *Xylocopa dejeanii*.

Characteristics	*X. dejeanii*
Genome assembly	
Size (Mb)	194.46
Number of scaffolds	141
Number of chromosomes	16
Scaffold N50 length (Mb)	13.15
GC (%)	43.50
BUSCO completeness (%)	99.0
Protein-coding genes	
Gene number	10,970
Mean gene length (bp)	6,715.5
BUSCO completeness (%)	99.0
Repetitive elements	
Size (Mb)	28.27 (14.54%)
DNA transposons (Mb)	5.85 (3.01%)
SINEs (kb)	14.13 (0.00%)
LINEs (Mb)	1.13 (0.58%)
LTRs (Mb)	5.61 (2.89%)
Unclassified (Mb)	7.84 (4.03%)
Number of ncRNA	432
rRNA	89
miRNA	67
snRNA	48

### Genome annotation

**Fig. 3:**
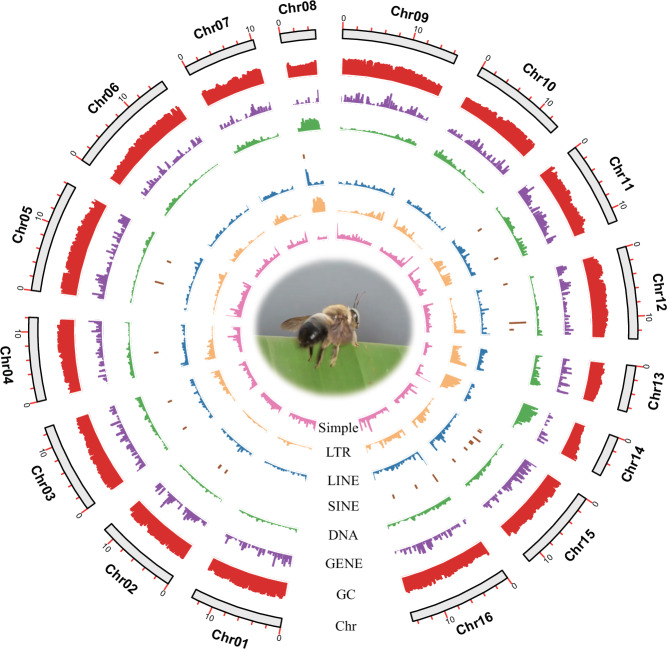
Circos plot showing the genomic characters of *Xylocopa dejeanii* from outer to inner: chromosome length, GC content, density of protein-coding genes, DNA transposons, SINE/LINE/LTR retrotransposons, and simple repeats.

RepeatModeler v2.0.2^23^ was employed to generate a *de novo* repeat database using the LTR discovery pipeline (-LTRstruct), which identifies specific repeat structures. This output database was subsequently merged with the Dfam 3.5^24^ and RepBase-20181026 databases^25^ to establish a custom library. RepeatMasker v4.1.2^26^ was performed to identify repetitive elements against our customized repeat library. The visual diagram of *X. dejeanii* genomic characteristics (LINE, LTR, DNA, SINE, GENE, GC, and Chr) were generated by TBtools^27^ (Fig. 3). We identified 14.54% (28.27 Mb) repetitive regions of the *X. dejeanii* genome, including 0.58% (1.13 Mb) long interspersed elements (LINEs), 2.89% (5.61 Mb) long terminal repeats (LTRs), 3.01% (5.85 Mb) DNA transposons, and 4.03% (7.84 Mb) unclassified (Table 3).

MAKER v3.01.03^28^ was utilized to predict protein-coding genes, which integrated evidence from *ab initio* predictions, transcript alignments, and homology-based approaches. BRAKER v2.1.6^29^ was performed to obtain *ab initio* gene prediction, which was applied to trained GeneMark-ES/ET/EP 4.68_lic^30^ and Augustus v3.4.0^31^, and integrating evidence from transcriptome and protein homology to accurately model sequence Properties. Hisat2 v2.2.0 was conducted to the transcriptome evidence in BAM alignments, and the reference proteins sourced from the OrthoDB v11^32^ database. For transcript prediction, the RNA-seq data were assembled with StringTie v2.1.6^33^, and genome assembly was used as reference^34^. Protein homology and intron position conservation in GeMoMa v1.9^35^ were used for predicting genes with the parameters “GeMoMa.c = 0.4 GeMoMa.p = 10”. Protein sequences from six insect species: *Anthidium xuezhongi* (GCA_022405125.1), *Apis mellifera* (GCF_003254395.2), *Bombus vancouverensis* (GCF_011952275.1), *Rhopalosiphum maidis* (GCF_003676215.2), *Tribolium castaneum* (GCF_000002335.3), *Solenopsis invicta* (GCF_016802725.1) were employed to increase search sensitivity. The outputs from GeMoMa and BRAKER were then integrated and provided as the *ab initio* input for MAKER.

In total, 10,970 protein-coding genes in the genome *X. dejeanii* were predicted, with an average length of 6715.5 bp. The number of introns, exons, and CDS per gene was 6.3, 7.4, and 6.9, respectively, and their mean lengths were 732.2, 355.8, and 252.5 bp, respectively. The BUSCO completeness assessment for protein sequences reached 99% (n = 1,367), reflecting the high quality of these predictions.

Gene functional annotation was performed using two distinct methods: the SwissProt and TrEMBL databases were searched via Diamond v2.0.8^36^ on “very sensitive” mode (e-value of 1e-5). InterProScan 5.41-78.0^37^ was used to predict protein domains by searching four public databases: SMART^38^, Pfam^39^, CDD^40^, and Superfamily^41^, InterProScan and eggnogmapper v2.1.5^42^ were conducted to annotate the Gene Ontology (GO) and pathway (KEGG, Reactome). Functional annotation attached 10,343 and 8,974 genes, matching the UniProtKB and InterProScan databases, respectively. Combining InterProScan and eggnog annotation results, we identified 8,461 GO items and 3,959 KEGG items.

Two approaches were used for ncRNA annotation: Infernal v1.1.4^43^ was performed to scan Rfam database to annotate rRNA, snRNA and miRNA; tRNAs were refined via tRNAscan-SE v2.0.9^44^, using the built-in script ‘Euk High Confidence Filter’ to filter out the low-confidence tRNAs. Totally, 432 ncRNAs were captured, including 198 tRNAs, 89 rRNAs, and 67 miRNAs. snRNAs were classed into 34 spliceosomal RNAs in eight groups (U1, U2, U3, U4, U5, U6, U11, and U12), two minor spliceosomal RNAs (U4atac, U6atac), eight C/D box snoRNAs, and one HACA-box snoRNA.

## Data Records

All raw data and the assembly of *X. dejeanii* have been submitted to NCBI under Project PRJNA977042, including transcriptome (SRR24955308)^45^, Hi-C (SRR24955309)^46^, Illumina (SRR24955310)^47^, and PacBio (SRR24955311)^48^ data. The NCBI accession number of the assembled genome is GCA_036983795.148^49^. Annotation results of repetitive and other gene predictions are submitted in the Figshare database^50^.

## Technical Validation

The genome quality of *X. dejeanii* was evaluated for completeness and accuracy. Genome completeness was analyzed using BUSCO, via the insects_odb10 database (n = 1,367), revealing 99.00% completeness, including 1,347 (98.50%) single-copy orthologs, seven duplicate BUSCOs (0.10%), two fragmented BUSCOS (0.1%), and 11 missing BUSCOs (0.90%). To assess assembly accuracy, we calculated the mapping rates of PacBio, Illumina, and RNA reads to the final assembly, and the mapping rates were 99.52%, 97.94%, and 91.80%, respectively. Manual correction was used for Hi-C assembly to ensure accuracy, and the heatmap revealed a highly structured pattern of interactions at the chromosomal level (Fig. 2).

## Data Availability

No specific script was used in this work. All bioinformatics analysis was performed according to relevant manuals, commands, and/or pipelines of the corresponding software packages.
